# 

**Published:** 1874-06

**Authors:** 


### CONTENTS:

### ORIGINAL COMMUNICATIONS.

ARTICLE I.—Protoplasm.—Its Relation to Vitality. By H. R. Noel, M. D., Professor of Physiology ........ 49

ARTICLE II.—The Use of Alcohol in Medical Practice. By John Morris, M. D........ 58

ARTICLE III —Mechanical Dentistry. By W. O. Kulp, D. D. S........ 64

### SELECTED ARTICLES.

ARTICLE IV.—Contour Fillings.—Has Experience Demonstrated their Practical Value. By H. L. Sage, D D. S ........ 67

ARTICLE V.—The Brain Power of Man By Brown-Sequard, M. D........ 82

ARTICLE VI—Cremation ........ 87

### EDITORIAL, ETC.

Bromide of Potassium in Convulsions and Dental Irritation........ 89

How New York Physicians and Dentists get a Competence ........ 89

The Agassiz Memorial........ 91

### MONTHLY SUMMARY.

Carbolic Acid in Carbuncle........ 92

Disease Destroying Tree........ 93

Extraordinary Malpractice ........ 94

Chloral Hydrate and Camphor in Neuralgia........ 94

Poisoning by Cantharidal Collodion........ 95

Remedy for Tooth-ache........ 95

A New Sign of Death........ 95

A Dentist Fined........ 96

Successful Case of Transfusion........ 96

Monstrosity ........ 96

A New Dental Hospital........ 96

Death from Chloroform and Hemorrhage........ 96

